# Compartmentalized Immune Response in Leishmaniasis: Changing Patterns throughout the Disease

**DOI:** 10.1371/journal.pone.0155224

**Published:** 2016-05-12

**Authors:** Alhelí Rodríguez-Cortés, Eugenia Carrillo, Susanna Martorell, Felicitat Todolí, Ana Ojeda, Alba Martínez-Flórez, Alicia Urniza, Javier Moreno, Jordi Alberola

**Affiliations:** 1 Departament de Farmacologia, de Toxicologia, i de Terapèutica, Universitat Autònoma de Barcelona, Bellaterra (Barcelona), Spain; 2 Leishmaniasis and Chagas Disease Unit, WHO Collaborating Centre for Leishmaniasis, Centro Nacional de Microbiologıía, Instituto de Salud Carlos III, Madrid, Spain; 3 Zoetis Inc., La Vall de Bianya, Girona, Spain; INRS - Institut Armand Frappier, CANADA

## Abstract

Visceral leishmaniasis (VL) is characterized by loss of T-cell responsiveness and absence of *Leishmania-*specific IFN-γ production by peripheral blood mononuclear cells. However, the expressions of IFN-γ and TNF-α are up-regulated in the tissues and plasma of VL patients. There is a paucity of information regarding the cytokine profile expressed by different target tissues in the same individual and the changes it undergoes throughout the course of infection. In this work we evaluated IFN-γ, TNF-α, IL-10, and TGF-β mRNA expression using real-time RT-PCR in 5 target tissues at 6 months and 16 months post-infection (PI) in a canine experimental model which mimics many aspects of human VL. The spleen and liver of *Leishmania infantum* experimentally-infected dogs elicited a pro- and anti- inflammatory response and high parasite density at 6 and 16 months PI. The popliteal lymph node, however, showed an up-regulation of IFN-γ cytokin at commencement of the study and was at the chronic phase when the IL-10 and TGF-β expression appeared. In spite of skin parasite invasion, local cytokine response was absent at 6 months PI. Parasite growth and onset of clinical disease both correlated with dermal up-regulation of all the studied cytokines. Our VL model suggests that central target organs, such as the spleen and liver, present a mixed cytokine immune response early on infection. In contrast, an anti-inflammatory/regulatory immune response in peripheral tissues is activated in the later chronic-patent stages of the disease.

## Introduction

In recent years, leishmaniasis has spread into formerly *Leishmania*-free ecoregions, becoming the third most frequent opportunistic parasitic disease in HIV patients [1,2]. Visceral leishmaniasis (VL) is caused by *L*. *donovani* and *L*. *infantum* and has a fatal outcome when let untreated. It is characterized by fever, hepatosplenomegaly, and cachexia which are thought to be associated with the absence of a specific cellular immune reponse (CMI) during active disease [3–5]. Proinflammatory cytokines (IFN-γ and TNF-α), however, increase in tissues such as the spleen, bone marrow (BM), lymph node (LN), and plasma [6–8] suggesting that instead of a suppressed T helper (Th)1 response, active VL might be due to unresponsiveness to Th1 stimuli. In this regard, IL-10 and TGF-β, cytokines known to suppress CMI, are increased in lesional tissues from human VL patients [7,9]. Knowledge concerning the cytokine VL profile in human target organs is still limited as it requires potentially dangerous and time-consuming invasive techniques.

Canine leishmaniasis (CanL) shares many characteristics of human VL, and the dog is the main peridomestic reservoir of *L*. *infantum* [10]. Protective responses in dogs have been associated with low anti-*Leishmania* antibody levels and the activation of specific CMI, including the production of Th1 cytokines, such as IFN-γ, TNF-α, and IL-2 [11,12]. On the other hand, active disease is characterized by a marked humoral response, specific immunossupression, and mixed Th1/Th2 cytokine profile [13–15]. Most research has, however, focused on the analysis of the immune response in peripheral blood even though it is currently accepted that the immune response to the parasite is not identical in the whole host system but rather organ-specific [reviewed by [16]]. This mixed Th1/Th2 cytokine profile is as yet not well defined because a number of studies evaluating different tissues obtained discrepant results [17–19]. In addition, whereas in human VL the role of IL-10 during active disease has been clearly established, in CanL its action is still controversial. Some authors have associated IL-10 with an increase in clinical signs [19,20] whilst others were unable to detect IL-10 accumulation during patent-phase disease [14,17]. Few studies have analyzed the immunological response in more than one target organ during CanL, and none at different times and compartments during disease manifestation.

The aim of the current study was to analyze the cytokine expression in immunological organs where antigen exposure and immune response take place, and also in organs targeted by the pathogen for active multiplication and expansion. Since cytokine levels were monitored at two different time points, we were able to gain unprecedented insight into how immunological reactions against *Leishmania* are coordinated. Moreover, we explored potential links amongst the spread of *Leishmania*, its progression, and the immune profile.

## Material and Methods

### Ethical statement

All procedures were approved by the Ethical Review Committee of Zoetis Manufacturing Research Spain S L [formerly known as Fort Dodge Veterinaria S.A.] (L'Hostalnou de Bianya, Girona, Spain) in compliance with national (Real Decreto 1201/2005) and European Union regulations (European Directive 86/609/CE) for projects using animals for research purposes. The project license number assigned by the Ethics Committee was number 132 and the permit number allocated by the Spanish Authorities was number 3303.

### Dogs and samples

Thirty-six, six-month-old, intact, female beagles weighing approximately 10 kg from Zoetis Manufacturing Research Spain S L [formerly known as Fort Dodge Veterinaria S.A.] (L'Hostalnou de Bianya, Girona, Spain) received routine vaccinations and were dewormed. They were housed in pens of six (~13 m² pen) or seven animals (~15 m² pen) under conditions designed to exclude natural *Leishmania* infection. They had direct contact with pen mates and neighboring pens, and received daily exercise in the facility corridors. There was environmental enrichment in the form of several toys and the interior of each cage had an elevated platform onto which the dogs could jump or hide below. None of the 36 dogs had detectable levels of anti-*Leishmania* antibodies in serum, or *Leishmania* DNA in BM and blood samples.

*L*. *infantum* (strain MCAN/ES/92/BCN-83/MON-1), obtained from a non-treated naturally infected dog, was passaged through hamsters in order to retain its full virulence. The spleens from infected hamsters were homogenized in Schneider’s *Drosophila* medium (Sigma, St Louis, MO, USA) supplemented with 20% v/v fetal calf serum (Gibco, Paisley, Scotland) and 25 μg·mL^−1^ gentamicin (Sigma), and centrifuged at 500 *g* for 10 min at 4°C. The resulting supernatant was washed three times and resuspended in 0.9% NaCl at a concentration of 2 × 10^8^ amastigotes·mL^−1^. Thirty-one dogs were immediately infected by intravenous injection of 1 mL of the resuspended parasites. The remaining 5 non-infected dogs were used as controls (NID). To minimize suffering, clinical end points for withdrawal were established based on clinical signs including severe skin lesions, marked lameness, debilitating diarrhea, neurological signs or critical bleeding from any orifice.

There were no deaths during the study period in any of the groups. Twenty-four infected dogs were euthanized at 6 months post-infection (PI) (Group 6-m) and 7 infected dogs at 16 months PI (Group-16m). The 5 NID dogs were euthanized at the end of the study. Prior to euthanasia, a complete physical examination was carried out and blood collected for clinical biochemistry analysis (renal and hepatic function tests, and protein electrophoresis). Clinico-pathological findings compatible with CanL were rated as previously described [21] and merged to obtain an overall clinico-pathological score (CPS) (Table 1). Euthanasia was carried out using a distress-free and painless protocol. First the dogs were anaesthetized with buprenorphine (Buprex^®^, Schering Plough) 10 μg/kg plus either medetomidine hydrochloride (Domtor^®^, Orion) 10 μg·kg^−1^ or acepromacine maleate (Calmo Neosan^®^, Pfizer) 0.5 mg·kg^−1^ administered intramuscularly. Approximately 30 minutes later they were euthanized with an intravenousoverdose (>200 mg·kg^−1^) of pentobarbital sodium (Dolethal^®^, Vetoquinol). Samples from the spleen, BM from the costochondral juntion, liver, popliteal LN, and healthy pinna skin were collected, snap-frozen in liquid nitrogen, and stored at −80°C until use.

**Table 1:** Clinicopathological findings in experimentally infected dogs at 6 months and 16 months post-infection.

Clinicopathological Findings	6 Month Group		16 Month Group	
	Number of Dogs (%)	Median Score	Number of Dogs (%)	Median Score
Weight loss	0	0	7 (100%)	1
Pale mucosa	0	0	5 (71%)	1
Lymphadenopathy	14 (58%)	1	7 (100%)	3
Ocular lesions	0	0	4 (57%)	1
Desquamation	0	0	6 (86%)	2
Alopecia	6 (25%)	0	7 (100%)	2
Skin ulcers	0	0	1 (14%)	0
Dermatitis	1 (4%)	0	4 (57%)	2
Nodular skin lesions	1 (4%)	0	1 (14%)	0
Mucocutaneous signs	0	0	1 (14%)	0
Gastrointestinal signs	0	0	1 (14%)	0
Lameness	0	0	1 (14%)	0
Clinical Score > 0	14 (58%)	1	7 (100%)	12
Clinical biochemistry	19 (79%)	1,2	7 (100%)	8,6
**Clinicopathological score**		**2.9**		**22.5**

### Serological tests

The dynamics of IgG and IgA against crude total *L*. *infantum* antigen (CTLA) in serum has been previously described [22]. Results were expressed as ELISA units (EU) in reference to a known positive serum used as a calibrator and arbitrarily set at 1 EU. Cut-off (mean + 3 *SD* for 32 dogs from non-endemic areas) was at 0.250 EU for IgG and 0.600 EU for IgA. Levels were categorized [23].

### Real-time PCR amplification of *L*. *infantum* DNA in tissue samples

DNA was extracted using High Pure PCR Template Kit (Roche). *L*. *infantum* DNA was specifically detected and quantified by Taqman qPCR (Applied Biosystems, Foster City, California, USA) as previously described [24] with minor modifications. Targeted qPCR conserved DNA regions of the kinetoplast minicircle DNA from *L*. *infantum* (LEISH-1 5’-AACTTTTCTGGTCCTCCGGGTAG-3’; LEISH-2 5’-ACCCCCAGTTTCCCGCC-3’ and TaqMan-MGB probe FAM-5’-AAAAATGGGTGCAGAAAT-3’- MGB). A standard curve was generated from *Leishmania* DNA extracted from 1 × 10^7^ parasites, using serial dilutions from 10^3^ to 10^−3^ parasites. Thermal cycling profile was 50°C for 2 min, 95°C for 10 min, 40 cycles at 95°C for 15 s, and 60°C for 1 min. Analyses were performed in a Step One Plus Real Time PCR System (Applied Biosystems Laboratories, Foster City, CA, USA). Each standard, sample, and a negative control was analyzed in triplicate for each run. The number of parasites per milligram of tissue was calculated and categorized [25]. This protocol was able to detect 0.001 parasites per reaction tube with a dynamic range of 10^7^. The median slope of three different standard curves was −3.44, and the qPCR efficiency of 98%. Quantification was linear between 10^3^ and 10^−2^ parasites per reaction tube (correlation = 0.99).

### Quantitative reverse transcription PCR (RT-qPCR) analysis of canine cytokines

Cytokine production was analyzed in tissues collected from the 5 NID dogs, 12 dogs from the 6-m group, and 7 from the 16-m group by RT-qPCR. Total RNA was extracted using RiboPure^™^ (Ambion). RNA samples were DNase treated with TURBO-DNA free Kit (Ambion) and quantified using NanoDrop-1000 UV-V Spectrophotometer, accepting ratios A260/A280 1.7–2.1. Each sample was stored at −80°C until use.

qPCR analyses for cytokine expression were performed with an Applied Biosystems ABI Prism 7000 DNA sequence detection system (PE Applied Biosystems, Foster City, CA, USA). Reverse transcription and qPCR amplifications were carried out in a single well with TaqMan PCR Core Reagent Kit (PE Applied Biosystems). The amplification conditions, gene-specific primers and probes were as previously described [26]. Parallel reactions were performed for canine IFN-γ, IL-10, TGF-β, and TNF-α transcripts from the tissues. Three replicate analyses were performed and the amount of target RNA was normalized with respect to the control (housekeeping) gene β-actin (ΔC). Results were expressed according to the 2^−ΔΔCt^ method using the mean value of the ΔC of the NID-group as calibrator.

### Statistics

Nonparametric statistical analysis was performed with the SPSS version 15.0 (SPSS, Inc., Chicago, IL). Differences between groups were analyzed by the Mann-Whitney *U* test, and correlations by Spearman's rank correlation coefficient. A *p*-value < 0.05 was employed.

## Results

### Clinical, serological, and parasitological course

**Fig 1:**
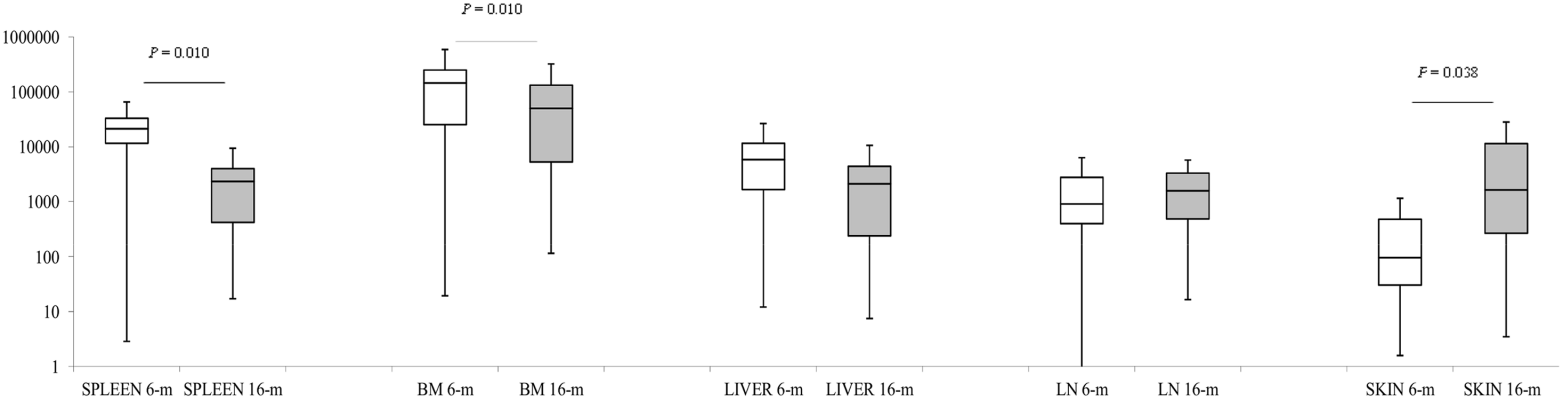
*L*. *infantum* parasite load detected at 6 months and 16 months post-infection in spleen, BM, liver, popliteal LN, and healthy pinna skin by real-time PCR. The number of parasites per milligram of tissue was calculated using a standard curve generated from DNA extracted from a known number of *L*. *infantum* promastigotes.

At 6 months PI the 24 evaluated dogs scored a CPS of 2.9 [1–5] (median [interquartil range]) with lymphadenopathy as the most frequent clinical sign (Table 1). *Leishmania*-specific IgG-CTLA seroreactivity was detected in all animals (145.08 EU [84.6–219.3]) but one. In contrast, only 9 of them presented *Leishmania*-specific IgA-CTLA seroreactivity (79.2 EU [51.7–138.2]). Parasites were detected in all the evaluated tissues for all the dogs from this group (Fig 1).

At 16 months PI all dogs showed symptoms and clinical chemistry compatible with leishmaniosis (Table 1), reaching a CPS of 22.5 [18–24], significantly different to that computed in dogs evaluated at 6 months (Mann-Whitney *U* test; *P* < 0.001). *Leishmania*-specific IgG-CTLA (300 EU [296.04–311.75]) and *Leishmania*-specific IgA-CTLA (119.4 EU [85.7–145.1]) seroreactivities were higher than those detected in dogs at 6 months PI, but only the increase in specific IgG levels was statistically significant (Mann-Whitney *U* test; *P* < 0.001). The parasite load increased in the skin between the two time points of the experimental infection (Mann-Whitney *U* test; *P* = 0.038). We also detected an increase in the LN and a decrease in the liver although these differences were not statistically significant. In contrast, the at 16 months PI the parasite load decreased in the spleen with respect to 6 months PI (Mann-Whitney *U* test; *P* = 0.010) (Fig 1).

### Course of cytokine levels in target organs

**Fig 2:**
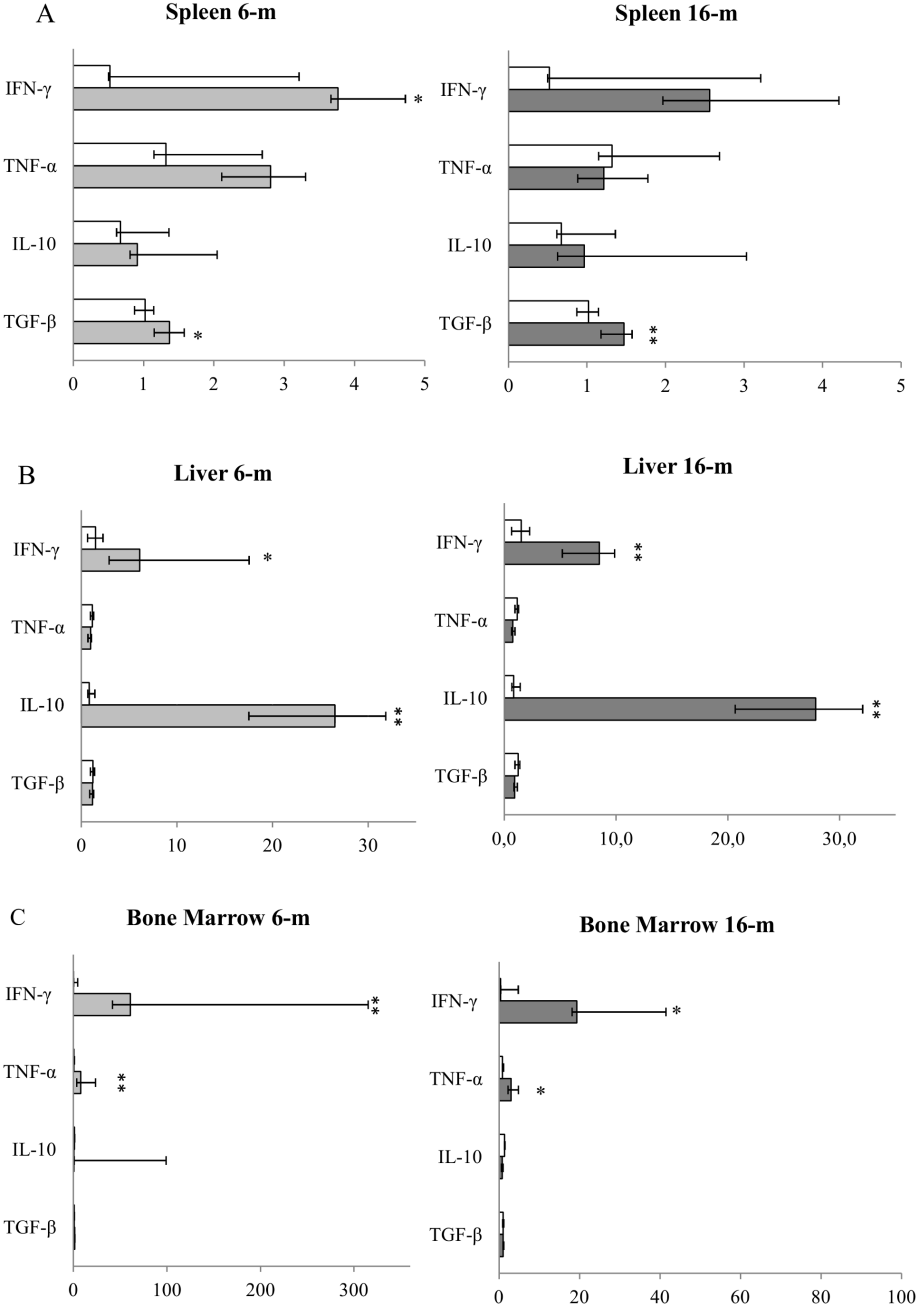
Median cytokine expression in (A) spleen, (B) liver, and (C) bone marrow (BM) samples throughout the course of *L*.*infantum* experimental infection. Y-axis: cytokine; X-axis: fold-increase of cytokine expression according to the 2^−ΔΔCt^ method using the mean value of the ΔC of the Non-infected dogs (NID) group as calibrator. White bars: NID; Bright-grey bars: dogs after 6 months (6-m) of *L*. *infantum* experimental infection; Dark-grey bars: dogs after 16 months (16-m) of *L*. *infantum* experimental infection. Significant differences in median cytokine levels among groups are indicated by * *P* < 0.05, ** *P* < 0.01, *** *P* < 0.001.

**Fig 3:**
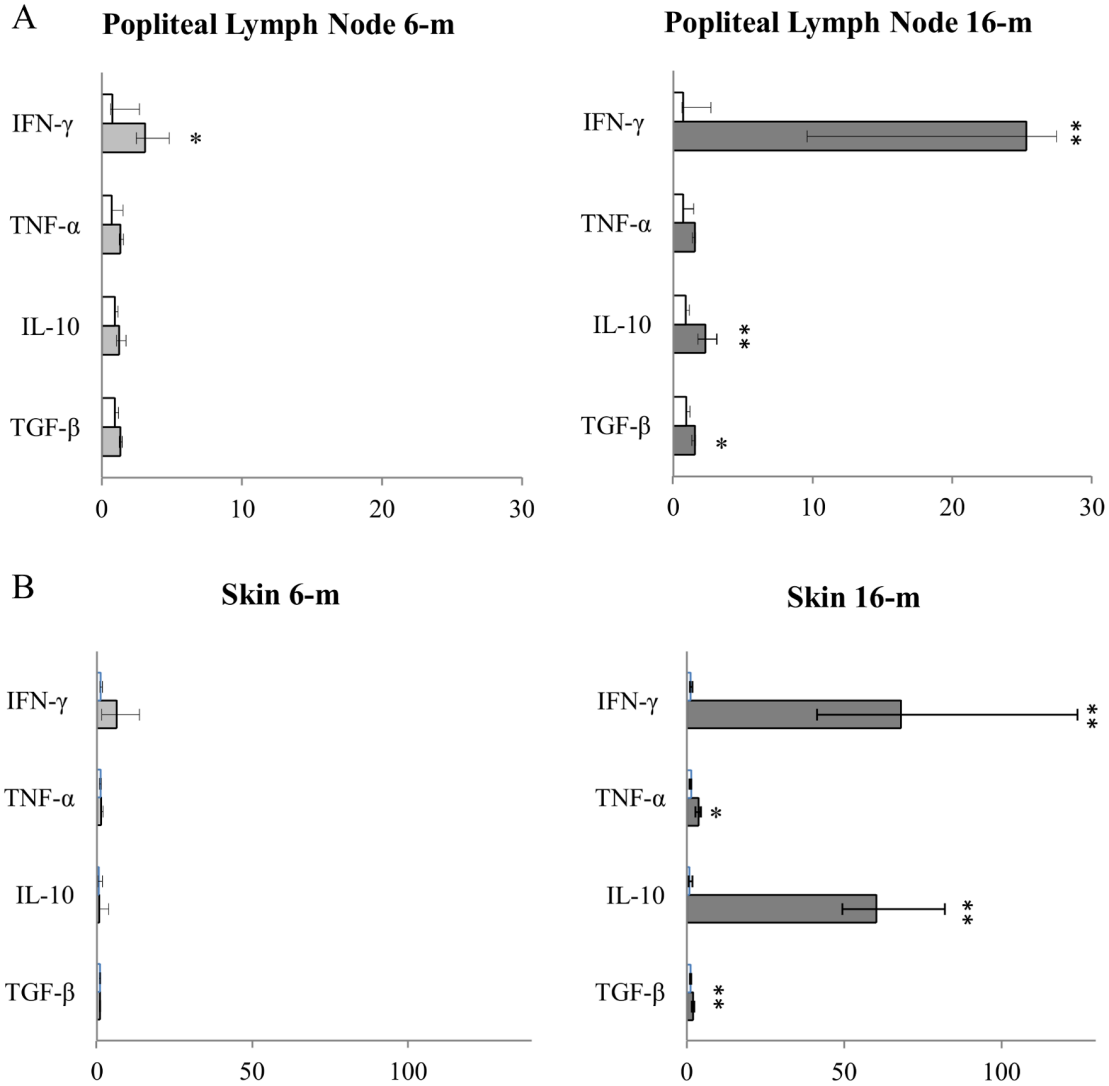
Median cytokine expression in (A) popliteal lymph node and (B) healthy pinna skin samples throughout the course of L.infantum experimental infection. Y-axis: cytokine; x-axis: fold-increase of cytokine expression according to the 2^−ΔΔCt^ method using the mean value of the ΔC of the Non-infected dogs (NID) group as calibrator. White bars: NID; Bright-grey bars: dogs after 6 months (6-m) of *L*. *infantum* experimental infection; Dark-grey bars: dogs after 16 months (16-m) of *L*. *infantum* experimental infection. Significant differences in median cytokine levels among groups are indicated by * *P* < 0.05, ** *P* < 0.01, *** *P* < 0.001.

mRNA levels for IFN-γ, TNF-α, IL-10, and TGF-β in the spleen, liver, BM, LN, and skin are shown in Figs 2 and 3.

At 6 months PI there was a statistically significant increase in the spleen (Mann-Whitney *U* test; *P* = 0.027), liver (Mann-Whitney *U* test; P = 0.040), BM (Mann-Whitney *U* test; *P* = 0.003), and LN (Mann-Whitney *U* test; *P* = 0.027) for IFN-γ when compared to the NID group. As for the other analyzed pro-inflamatory cytokine, TNF-α, only BM showed a statistically significant increase (Mann-Whitney *U* test; *P* = 0.003) when compared to the NID group. With regard to anti-inflammatory/regulatory cytokines, there was a statistically significant increase in the spleen (Mann-Whitney *U* test; *P* = 0.037) for TGF-β and in the liver (Mann-Whitney *U* test; P = 0.040) for IL-10 when compared to the NID group. It is worth highlighting that despite the presence of parasites in the skin no cytokine expression was detected at 6 months PI.

At 16 months PI, when all the infected dogs presented patent leishmaniosis, the statistically significant increase in IFN-γ was maintained in the liver (Mann-Whitney *U* test; *P* = 0.006), BM (Mann-Whitney *U* test; *P* = 0.043), and LN (Mann-Whitney *U* test; *P* = 0.003) when compared to the NID group. This was also the case for TGF-β in the spleen (Mann-Whitney *U* test; *P* = 0.010) and for IL-10 in the liver (Mann-Whitney *U* test; *P* = 0.010).

In LN there was a statistically significant increase in TGF-β (Mann-Whitney *U* test; *P* = 0.018) and IL-10 (Mann-Whitney *U* test; *P* = 0.003) when compared to NID; whereas previously, at 6 months, there had been only mRNA expression for IFN-γ. Finally, and in contrast to what occurred at 6 months PI, in the skin there was a marked expression of all the analyzed cytokines when compared to the NID group: IFN-γ (Mann-Whitney *U* test; *P* = 0.003), TNF-α (Mann-Whitney *U* test; *P* = 0.018), IL-10 (Mann-Whitney *U* test; *P* = 0.010), and TGF-β (Mann-Whitney *U* test; *P* = 0.003).

### Relationships between tissue cytokine expression and markers of *Leishmania* infection

The relationships between tissue cytokine expression and markers of *Leishmania* infection are summarized in Table 2.

**Table 2:** Relationships amongst cytokine expression in different target organs and clinicopathological score, *Leishmania*-specific IgG and IgA levels, and parasite load.

TISSUE	CK	Clinico-pathological score		*Leishmania*-specific IgG levels		*Leishmania*-specific IgA levels		Parasite Load	
		ρ^a^	*P*	ρ	*P*	ρ	*P*	ρ	*P*
SPLEEN	IFN-γ	0.197	NS^b^	0.122	NS	0.271	NS	0.542	0.008
	IL-10	0.177	NS	0.041	NS	-0.073	NS	0.397	NS
	TGF-β	0.459	0.024	0.343	NS	0.353	NS	0.557	0.006
	TNF-α	-0.315	NS	-0.258	NS	-0.26	NS	0.153	NS
LIVER	IFN-γ	0.392	NS	0.528	0.014	0.655	0.001	0.47	0.027
	IL-10	0.450	0.036	0.446	0.043	0.345	NS	0.373	NS
	TGF-β	-0.228	NS	0.03	NS	0.035	NS	0.045	NS
	TNF-α	-0.188	NS	-0.033	NS	0.1	NS	0.02	NS
BM^c^	IFN-γ	0.396	NS	0.309	NS	0.582	0.018	0.632	0.011
	IL-10	-0.201	NS	-0.246	NS	-0.208	NS	-0.348	NS
	TGF-β	0.138	NS	-0.05	NS	0.25	NS	0.407	NS
	TNF-α	0.455	NS	0.314	NS	0.584	0.017	0.624	0.013
LN^d^	IFN-γ	0.626	0.001	0.746	<0.001	0.537	0.008	0.302	NS
	IL-10	0.561	0.004	0.584	0.003	0.447	0.033	0.238	NS
	TGF-β	0.514	0.01	0.432	0.039	0.381	NS	0.364	NS
	TNF-α	0.236	NS	0.312	NS	0.415	0.049	0.12	NS
SKIN	IFN-γ	0.817	<0.001	0.810	<0.001	0.644	0.001	0.596	0.003
	IL-10	0.656	0.001	0.656	0.001	0.492	0.02	0.558	0.006
	TGF-β	0.584	0.003	0.599	0.003	0.439	0.041	0.386	NS
	TNF-α	0.711	<0.001	0.655	0.001	0.490	0.021	0.684	<0.001

^a^ Spearman coefficient of correlation

^b^ non significant (*P* > 0.05)

^c^ Bone Marrow

^d^ Popliteal Lymph Node

A positive correlation was found in the spleen between parasite load and both IFN-γ and TGF-β expression. TGF-β was the only cytokine whose expression correlated with CPS. It should be noted that no correlation was found between cytokines and *Leishmania*-specific IgG or IgA antibody levels in this tissue.

When we evaluated the relationship between different parameters in liver samples, we found that IFN-γ correlated with both parasite load and *Leishmania*-specific IgG and IgA antibody levels. IL-10, however, was the only cytokine whose expression correlated with CPS in this organ.

BM showed that expression of IFN-γ and TNF-α correlated with parasite load and *Leishmania*-specific IgA.

IFN-γ, IL-10, and TGF-β were positively correlated with CPS and *Leishmania-*specific IgG levels in LN. Interestingly, popliteal LN was the only tissue where no cytokine correlated with the parasite load.

There was a positive correlation in the skin among all cytokines and all markers of *Leishmania* infection, with the exception of TNF-α levels and dermal parasite density.

**Fig 4:**
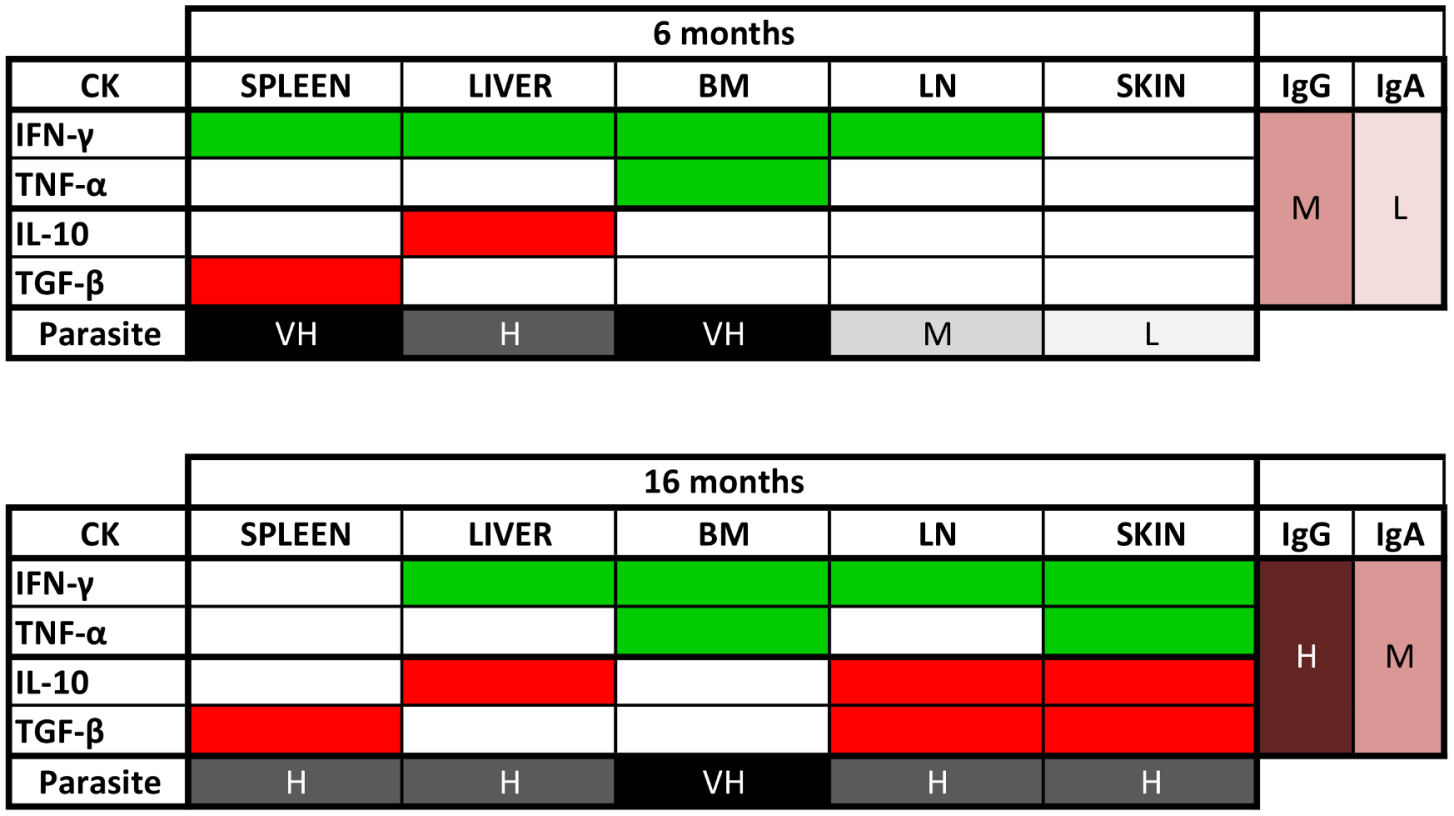
Graphical representation of cytokine activation, parasite load, and *Leishmania*-specific antibody levels at 6 and 16 months PI. Cytokines: Green = activation of pro-inflammatory cytokines (statistically different from NID). Red = activation of anti-inflammatory/regulatory cytokines (statistically different from NID). Parasite load was categorized as low (L: 1–100 parasites mg^-1^ of tissue), medium (M: 101–1000), high (H: 1001–10,000), and very high (VH > 10,000). Antibody levels (*Leishmania*-specific IgG and IgA) were categorized as low (L: 1 × cut-off), medium (M: 2 × cut-off), and high (H: 3 × cut-off).

All the results obtained in the present study are summarized in Fig 4.

## Discussion

*L*. *infantum* is an obligatory, intracellular parasite with tropism towards macrophages in specific organs such as the spleen, liver, BM, LN, and skin. To date, most immunological studies have either focused on the analysis of the immune response in peripheral blood or have only examined one tissue or time point during *Leishmania* infection. The present study evaluated the immune response and parasite load of different organs in 6-month experimentally-infected dogs that were still clinically healthy, and in 16-month experimentally-infected dogs suffering from the patent phase of the disease. Our aim was to establish relationships amongst the spread of *Leishmania*, its progression, and the immune profile.

It is clear from our data that *L*. *infantum* experimental infection in dogs leads to an increased expression of IFN-γ in most target organs which is maintained in later stages of the disease. Other studies, in both experimentally and naturally infected dogs, have already reported an augmentation for some tissues such as the spleen [27], liver [28], BM [17], and LN [29] during the patent phase of the disease. Until recently, however, there has been no evidence that this pro-inflammatory response is widespread and positively correlated with the parasite load for each organ or tissue. Our results are in accordance with previous studies carried out in hamsters and humans suffering from VL where high parasite loads were observed even in pro-inflammatory environments [6,30,31]. IFN-γ production during the patent phase rules out a Th1 inherent defect although its expression is not enough to confer full protection, as has already been observed in human VL [32]. In our study, it is probable that most of the dogs expressed IFN-γ in response to the presence of parasites in the target organs, and this inflammatory response persisted due to an overreaction against persistent infection [33]. Our findings raise the question of whether the expression of IFN-γ in the 16-month group exacerbated the clinical signs and was actually involved in CanL immunopathology. A similar phenomenon has been observed in human mucocutaneous leishmaniasis [34] and other parasitic diseases such as malaria [35] and Chagas disease [36]. A number of authors have provided contradictory results after analyzing peripheral blood mononuclear cells from infected dogs which did not produce IFN-γ [11]. Nevertheless, our work demonstrates that in experimental CanL the immune system is greatly stimulated in target organs such as the spleen and liver, and it is possible that other factors may counteract its leishmanicidal action.

IL-10 and TGF-β are macrophage deactivating cytokines which play key roles in murine and human VL [37,38]. In the present study, we found that experimental infection with *L*. *infantum* led to significantly increased IL-10 and TGF-β expression in the liver and spleen, respectively. These two cytokines are produced by various cell types, including macrophages, B lymphocytes, and CD4+ T cells, and exert their immunosuppressive action by suppressing macrophage activation, dendritic cell migration, and protective Th1 responses [33]. Co-expression of pro- and anti-inflammatory cytokines in the spleen and liver, a mixed Th1/Th2 pattern, has been previously described. This relationship suggests that an equilibrium between the two types of responses is required in order to control the disease [27,39]. In the present study, we did not observe significant differences in the expression of any of the evaluated cytokines in the spleen or liver when comparing clinically healthy, infected dogs with sick ones. A finding that was probably due to the high parasite load present in these organs in both groups of dogs and which implies a very strong immune activation. As a consequence, our results suggest that, in this model, measuring the expression of cytokines in the spleen or liver serves no purpose from a prognostic point of view.

To the best of our knowledge, there are only two reports on the expression of cytokines in the BM of dogs suffering from leishmaniasis [17,29]. In agreement with our results, they conclude that BM tends to develop high parasite loads in a predominantly pro-inflammatory environment, characterized by an increased expression of IFN-γ and TNF-α, and no detection of IL-10. The augmentation of TNF-α in BM, the highest detected in our study, may have been due to the stimulation of myelopoiesis as previously described in *L*. *infantum* infected dogs [40] and *L*. *donovani* infected mice [41]. BM tissue could provide progenitor cells to the spleen that activate and contribute to granuloma formation [42,43].

A major finding of our study is the different pattern of cytokine expression observed when comparing skin and LN with the other analyzed tissues. At 6 months PI no significant increase in cytokine expression in skin was found and only an up regulation of IFN-γ was detected in LN. These results show for the first time that there is a silent phase in the skin: parasite invasion takes place without cytokine expression, similar to that which occurs in *L*.*major* infected mice [44]. This silent phase may allow *Leishmania* to survive and settle in the dermis without the presence of a local immune response that would lead to parasite elimination. Such a lack of immune response could play a major role in the transmission cycle of the parasite and might explain why clinically healthy, naturally-infected dogs are infective to phlebotomines [45]. Our results indicate that the phase of disease control during experimental infection was characterized by a pro-inflammatory immune response in peripheral LN in conjunction with a silent phase in the skin.

In contrast, there was a sharp increase of expression of anti-inflammatory/regulatory TGF-β and IL-10 cytokines at 16 months PI in LN and skin which paralleled the increased parasite load in these tissues. Furthermore, there was also a major inflammatory response in the skin. The skin and LN were the only tissues where the expression of all the cytokines correlated positively with the clinical score and with *Leishmania*-specific IgG and IgA (with the exception of TNF-α in LN). Our results not only confirm the association of IL-10 and TGF-β expressed in skin and LN with clinical signs [16,18,46], but also, for the first time, they can be compared with the immune response in other target organs. The fact that the expression of anti-inflammatory/regulatory cytokines takes place only in the skin and popliteal LN of 16-month infected dogs is possibly due to the organs’ peripheral nature. Following an intravenous administration of the parasite they are invaded later than the liver, spleen, and BM [47]. If the parasites can not be contained by the central target organs they then spread towards the popliteal LN and skin where they increase their load and activate local immune pro- and anti-inflammatory responses. The high level of skin IL-10 expression found at 16 months PI, the greatest out of all the analyzed tissues, is noteworthy. At this point it is difficult not to draw a parallel between that which takes place in *Leishmania*- sick dogs and human post-kala-azar dermal leishmaniasis (PKDL). Differences aside, in the two instances there are skin lesions produced by a *Leishmania* spp. with visceral tropism and high local IL-10 expression, and both the affected humans and dogs play a paramount role in the transmission of the parasite [48]. It would be of interest to compare the healthy and lesional skin of both dogs and humans suffering from PKDL.

In short, our study shows that IFN-γ expression is present in most tissues at both 6 and 16 months PI. Nevertheless, the anti-inflammatory/regulatory immune response at 6 months PI typically only occurs in the spleen and liver which is when the dogs are still clinically healthy but carry high parasite loads in these organs. At 16 months PI, the immune response, characterized by the expression of IL-10 and TGF-β, is highly activated in popliteal LN and skin, paralleling both the increase in parasite load and the appearance of clinical signs. From our findings we postulate that sequential parasite dissemination, in tandem with a sequential deactivating immune response, occurs in target organs in this CanL experimental model. Our study contributes to characterizing this animal model in VL for vaccine research and should help to identify new targets and strategies to fight this neglected tropical disease.
